# Update on the role of extracellular vesicles in rheumatoid arthritis

**DOI:** 10.1017/erm.2021.33

**Published:** 2022-03-17

**Authors:** Hai-bing Miao, Fang Wang, Shu Lin, Zhen Chen

**Affiliations:** 1 Department of Rheumatology and Immunology, The Second Affiliated Hospital of Fujian Medical University, Quanzhou, Fujian, China; 2 Centre of Neurological and Metabolic Research, The Second Affiliated Hospital of Fujian Medical University, Quanzhou, Fujian, China; 3 Diabetes and Metabolism Division, Garvan Institute of Medical Research, 384 Victoria Street, Darlinghurst, Sydney, NSW 2010, Australia

**Keywords:** Exosomes, extracellular vesicles, immunomodulation, mesenchymal stem cell-derived extracellular vesicles, rheumatoid arthritis

## Abstract

Rheumatoid arthritis (RA) is a heterogeneous autoimmune disorder that leads to severe joint deformities, negatively affecting the patient's quality of life. Extracellular vesicles (EVs), which include exosomes and ectosomes, act as intercellular communication mediators in several physiological and pathological processes in various diseases including RA. In contrast, EVs secreted by mesenchymal stem cells perform an immunomodulatory function and stimulate cartilage repair, showing promising therapeutic results in animal models of RA. EVs from other sources, including dendritic cells, neutrophils and myeloid-derived suppressor cells, also influence the biological function of immune and joint cells. This review describes the role of EVs in the pathogenesis of RA and presents evidence supporting future studies on the therapeutic potential of EVs from different sources. This information will contribute to a better understanding of RA development, as well as a starting point for exploring cell-free-based therapies for RA.

## Introduction

Rheumatoid arthritis (RA) is a widespread chronic immune-mediated disease characterised by progressive symmetric polyarthritis (Ref. 1). The exact mechanism of RA has not been elucidated yet, but distinct mechanisms such as gene–environment interactions, immune disorders and stromal tissue disorders have been proposed (Ref. 2). During the past few decades, various treatments for RA have been suggested. In particular, the application of effective biological and small molecule kinase inhibitors has substantially improved the clinical efficacy of RA treatment. However, it is important to consider the toxic effects associated with the chronic use of these drugs. Additionally, many patients treated with these agents do not show diminished joint and systemic inflammation (Ref. 3), making a case for more effective RA treatment strategies.

Extracellular vesicles (EVs) represent a heterogeneous group of membrane-enclosed vesicles originating from different types of cells (Ref. 4). EVs are particles naturally secreted by cells, with excellent stability and biocompatibility and low toxicity and immunogenicity, and their surface proteins reflect those of the parent cell (Refs 4, 5). They can reach the targeted cells and transfer their cargoes through cellular uptake. This process may trigger a functional response (Refs 6, 7). Numerous EVs have been detected in the circulating and synovial fluids of RA patients, prompting the investigation of their role in the pathogenesis of RA (Ref. 8). In this review, we summarise the existing research on EVs in RA, with emphasis on exosomes, and discuss their role in and therapeutic potential for RA.

## Rheumatoid arthritis

RA is a systemic and heterogeneous inflammatory autoimmune disease characterised by persistent synovitis, as well as cartilage and bone damage, which ultimately leads to irreversible joint deformities. Although the major histocompatibility complex (MHC) *HLA-DRB1* gene is considered the strongest genetic risk factor for RA, the exact pathogenesis of this disease remains unknown (Ref. 9). Systemic immune dysregulation, such as T helper (Th) 1/Th2 and Th17/regulatory T (Treg) cell imbalance, appears to play a critical role in the pathogenesis of RA, although some studies have suggested that Treg dysfunction is unrelated to the initiation of RA but is affected instead by the local inflammatory environment (Refs 10–13). Moreover, T cells can affect immunoglobulin (Ig) conversion, which may be related to autoantibodies. Follicular T helper cells, a subset of CD4+ T cells, are involved in B cell activities, including the generation of live plasma cells and memory B cells, promoting Ig affinity maturation, and stimulating B cell responses in RA (Ref. 14). In short, pathogenic T cells contribute to dysfunction of both the cellular and humoral responses in RA, partially reflecting the destruction of self-tolerance and the emergence of autoimmunity.

During RA development, various immune cells, including T cells, B cells and other innate cells, infiltrate the synovial membrane, and the levels of inflammatory factors increase. Invading T cells that undergo pyroptosis can trigger tissue inflammation and remodelling and might play a role in the chronic nature of synovitis (Refs 15, 16). Fibroblast-like synovial cells (FLSs) interact with these inflammatory components, lose their contact inhibition potential, downregulate cell apoptosis and become functionally transformed into pro-inflammatory effector cells, further prolonging and aggravating the inflammation of the synovial membrane. They also actively promote the flow of immune cells and express a variety of inflammatory cytokines, mediators and extracellular proteases, thereby exacerbating the pathogenesis of RA (Refs 17–19). Furthermore, FLS-derived interleukin 6 (IL-6) promotes the transformation of Foxp3 + CD4+ T cells into Th17 cells, which are more capable of inducing osteoclast production than any other T cell subset (Ref. 20). Additionally, bone erosion is promoted by antibodies against citrullinated proteins and abnormally elevated concentrations of cytokines, such as IL-1, IL-6, IL-17 and tumour necrosis factor alpha (TNF-*α*) (Ref. 18). Macrophages also play an important role in RA. It has been reported that the M1/M2 ratio is higher in RA synovial fluid, indicating that macrophages are polarised towards a pro-inflammatory phenotype (Ref. 21). These macrophages are one of the main sources of TNF-*α*, which is an important enhancer of osteoclastogenesis (Ref. 21). Briefly, the complex network of pathogenic mediators, including FLSs and immune cells, as well as the abnormal levels of cytokines and signalling molecules (IL-1, IL-6, TNF-*α*, IL-17, etc.), help induce persistent synovitis and joint destruction.

## Extracellular vesicles

EVs are a heterogeneous group of vesicles released by all types of cells, which cannot replicate on their own. The names of EV subtypes mentioned in the literature are not consistent (including exosomes, nanovesicles, microvesicles, microparticles, ectosomes, oncosomes and many others). Here, we considered EVs to include exosomes and ectosomes, and designated those <200 nm as small EVs (sEVs) in accordance with The International Society for Extracellular Vesicles 2018 (Refs 22, 23).

### Biogenesis and characteristics of EVs

**Fig. 1.**
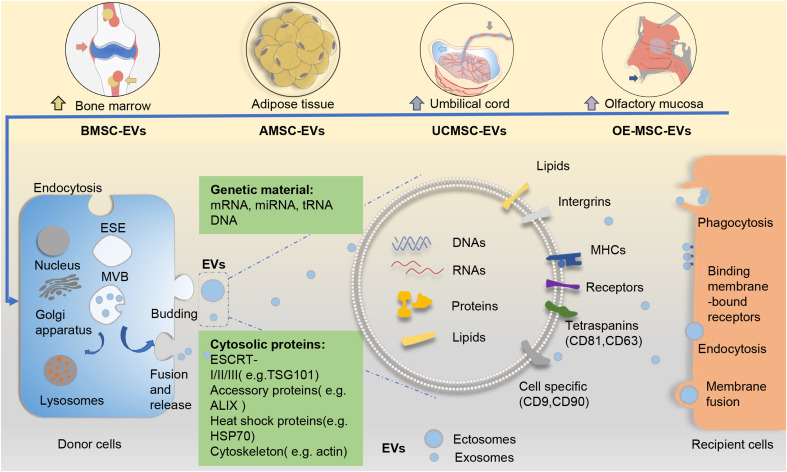
EVs from different sources of mesenchymal stem cells and their biogenesis pathways. The biogenesis of EVs (including exosomes and ectosomes) from different sources of MSCs follows a general process of EV production. Generally, ectosomes derive from direct budding from the plasma membrane, whereas exosomes result from two invaginations of the plasma membrane. EVs arrive at recipient cells and elicit functional responses via cellular uptake. EVs contain abundant cargoes (including DNAs, RNAs, proteins, lipids, etc.). Several surface molecules such as CD9, CD81 and CD63 can help to identify the origins of EVs from MSCs. ALIX, apoptosis-linked gene 2-interacting protein X; AMSC-EVs, adipose tissue mesenchymal stem cell-derived extracellular vesicles; BMSC-EVs, bone marrow mesenchymal stem cell-derived extracellular vesicles; ESCRT, endosomal sorting complexes required for transport; EVs, extracellular vesicles; HSP70, heat shock protein 70; MHC, major histocompatibility complex; MSC-EVs, mesenchymal stem cells; OE-MSC-EVs, olfactory ecto-mesenchymal stem cell-derived extracellular vesicles; TSG101, tumour susceptibility gene 101; UCMSC-EVs, umbilical cord mesenchymal stem cell-derived extracellular vesicles.

Generally, EVs can be divided into two major categories based on their biogenesis (Fig. 1). Ectosomes, which include microvesicles and microparticles and range from ~50 nm to 1 μm in diameter, bud directly from the plasma membrane. Exosomes are smaller, ranging from 40 to 160 nm (~100 nm on average), originate from the endosomal pathway, and usually express CD63, CD81 and CD9 on their surface (Ref. 23). Unlike that for ectosomes, exosome biogenesis normally involves two invaginations of the plasma membrane. The first invagination induces cup-shaped vesicles, which form early endosomes and then mature into late sorting endosomes. The second inward invagination involves the endoplasmic membrane and leads to the formation of intracellular multivesicular bodies containing intraluminal vesicles. After the intracellular multivesicular bodies fuse with the plasma membrane, the intraluminal vesicles are released through exocytosis as the final exosomes (Refs 4, 24). In terms of physical characteristics, there is overlap between ectosomes and exosomes, and it remains difficult to assign EVs to specific biological pathways in practice (Ref. 22). Dennis *et al*. suggested that membrane-associated annexin A1 is a potential marker specific for ectosomes that can distinguish them from exosomes (Ref. 25). In addition, endosomal sorting complexes required for transport, along with accessory proteins such as tumour susceptibility gene 101 (TSG101), and apoptosis-linked gene 2-interacting protein X have been implicated in the origins of EVs and the pathways of biogenesis (Ref. 26). Some of these molecules have been used as biomarkers for EVs (Ref. 27).

There is presently no consensus on the classification of EVs. Exploring the mechanism underlying the biogenesis of EVs could help identify potential EV subtype-specific markers and thus enable a more accurate characterisation of EVs.

### Cargoes and functions of EVs

EVs have key roles in cell-to-cell communication. Studies have indicated that EVs can transfer their contents to elicit functional responses, and several studies have shown that they can mediate signalling pathways through surface receptor contact between cells (Refs 28–30). EVs contain thousands of different bioactive molecules, including surface proteins, intercellular proteins, amino acids, metabolites, mRNAs, non-coding RNA species and DNA (Refs 25, 31, 32). EVs play an active role in different processes, such as angiogenesis, antigen presentation, cellular homoeostasis, inflammation and immunomodulation (Refs 30, 33–35). Exosomal microRNAs (miRNAs) have been shown to act as biomarkers and mediators in the pathophysiology of various diseases (Refs 35, 36). As natural particles secreted from cells, EVs possess excellent stability and biocompatibility and low toxicity and immunogenicity, making them promising next-generation drug candidates (Refs 5, 37).

Technologies and methodologies for the study of EVs are being constantly improved. Previous studies have reported that double-stranded DNA (dsDNA) and dsDNA-binding histones are related to EVs (Refs 31, 32, 38). However, a recent study used stepwise high-resolution density gradients and direct immunoaffinity capture to characterise the materials in the EV and the nanoparticle component. The results showed that no dsDNA or DNA-binding histones was detected in the sEV fractions, suggesting that sEVs are not vehicles of active DNA release (Ref. 25). In addition, the study further demonstrated that many of the presumed components of exosomes (such as annexin A2, histones, the glycolytic enzyme GAPDH, etc.) were absent from classical exosomes expressing CD63, CD81 and CD9 (Refs 25, 32). This shows that the precise identification of the molecular component of EVs needs to be further improved.

### Mesenchymal stem cell-derived EVs and immunomodulation

Mesenchymal stromal cells (MSCs) are a class of heterogeneous stem cells capable of self-renewal and multipotency. They can be obtained from many types of tissues, such as bone marrow, adipose tissue, umbilical cord, placenta, gingival tissue, periosteum and synovium (Refs 39–41) (Fig. 1). MSCs have been widely investigated in autoimmune diseases, including RA, owing to their immunomodulatory and regenerative properties. Recently, EVs derived from different sources of MSCs have been explored as a novel strategy for cell-free therapy in various diseases, including RA (Refs 35, 42–44). Based on current studies, EVs of MSCs have been demonstrated to be one of the important mediators for MSCs to exert their functions, and can perform the indispensable functions of their parental cells (Refs 42–45).

The immunomodulatory capacity of MSC-derived EVs (MSC-EVs) extends the effect of their parental cells to various effector cells in both innate and adaptive immunity (Refs 46–49). Generally, MSC-EVs suppress T cell proliferation and activation, regulate T cell differentiation and modulate the balance of Th1/Th2 and Treg/Th17 cells (Refs 43, 46, 47). In addition, MSC-EVs inhibit B cell proliferation and produce Ig (Refs 47, 49). MSC-EVs also modulate innate immune activities, such as macrophage polarisation towards the M2-like phenotype and the maturation of dendritic cells (DCs), which is required for the induction of effector T cells (Ref. 48).

However, the impact of MSC-EVs on the immune response may be inconsistent in EVs from different origins and different experimental context (Refs 50–52). One study reported that human bone marrow mesenchymal stem cells (BMSCs) isolated by ultracentrifugation and identified by flow cytometry had lower effects on T cell proliferation and plasma cell antibody formation than their parental cells (Ref. 52). Also, EVs from human adipose tissue-derived mesenchymal stem cells confirmed by cryo-electron microscopy cannot reduce the number of memory B cells and induce regulatory B cells (Ref. 51). Through the separation of EVs from the supernatant of urine stem cells by ultracentrifugation, and the identification of their characteristics by transmission electron microscopy, nanoparticle tracking and western blotting, Zidan *et al*. showed that these EVs could stimulate the differentiation of B cells and the production of IgM antibodies. Similar results were obtained when the experiment was repeated with purified B cells. Inside MSC-EVs, the authors reported the presence of B-cell activating factor and a proliferation-inducing ligand, as well as IL-6 and CD40L, all of which play central roles in B cell stimulation (Ref. 50).

The contradictory findings of these studies may result from the different sources of EVs and the consequent difference in the abundance of cargo in the vesicles. Some of the differences can also be explained by the different methodologies involved, including EV purification and quantitative methods. Further evidence will be needed to clarify the role of EVs in relation to their origins and experimental context.

## Role of EVs in the pathogenesis of RA

EVs were found to be significantly more abundant in the circulation and synovial fluid of RA patients than in those of healthy controls or patients with other types of inflammatory arthritis such as osteoarthritis (Refs 8, 53). EVs, and particularly exosomes, are internalised by the recipient cells and play an important role in the pathogenesis of RA by transferring their contents and regulating cell signalling pathways. The origin of EVs in the circulation and synovial fluid of RA patient remains unclear. Most studies have shown that platelets are the main source of EVs. In addition, evidence points also to monocytes, lymphocytes, red blood cells and local stromal cells and tissue cells (Ref. 8).

### EVs are involved in the immunopathology of RA

EVs from the circulation and synovial fluid can dysregulate T cell proliferation and differentiation, disrupt the Th17/Treg balance and alter the levels of inflammatory cytokines. The exosomal miR-17 level is upregulated in circulating exosomes of RA patients and inhibits Treg differentiation by suppressing the expression of transforming growth factor beta (TGF-*β*) receptor II (Ref. 54). An important pathological feature of RA is the hypoxic microenvironment (Ref. 55). When the exosomes of FLSs in an RA model are exposed to hypoxic conditions in vitro, the level of exosome miR-424 increases. Furthermore, exosomal miR-424 negatively regulates the expression of FOXP3 and increases the levels of pro-inflammatory cytokines IL-17, IL-22, IL-1*β* and TNF-*α* in RA mice (Ref. 56) (Table 1). In addition to changes in the distribution of miRNAs in EVs, surface molecules are also involved in the pathogenesis of RA. Programmed death 1 (PD-1) is an inhibitory molecule that regulates T cells (Ref. 57). A recent study showed that EVs in the plasma and synovial fluid of RA patients express PD-1 receptor and can transfer it to co-cultured lymphocytes. Although, co-culture of EVs and lymphocytes showed that transferring PD-1 could not reverse the proliferation of T cells induced by EVs (Ref. 58). Interestingly, one previous study identified the enhanced expression of PD-1 in the synovium of RA patients and that PD-1-induced suppression of T cell proliferation and production of cytokines such as interferon-*γ* (IFN-*γ*) were impaired by synovial inflammation (Ref. 59). This evidence suggests the insufficient effect of EV-transported PD-1, which deserves further investigation. TNF-*α* is a vital cytokine involved in the development of RA. Zhang *et al*. showed that TNF-*α* on exosomes from FLSs of RA patients (RA-FLS-exos) might affect T cell activation-induced cell death and render these activated T cells resistant to apoptosis (Ref. 60). In addition, several molecules on EVs can be recognised as autoantigens after citrullination in RA, thereby participating in formation of the immune complex (Ref. 61). Therefore, EVs might drive the pathogenesis of RA through immune complex-mediated pathways. A recent study showed that circulating EVs, mainly immune complex-EVs from seropositive RA, could activate monocytes and stimulate the release of inflammatory factors IL-1*β*, IL-6 and TNF-*α* (Ref. 61). In another study, the co-culture of monocyte-derived macrophages with circulating immune complex-EVs from RA patients revealed the M1-like profile of macrophages, thus enhancing T cell proliferation and significantly decreasing the frequency of dead B cells in co-cultures (Ref. 62). In summary, EVs participate in various immune activities related to RA development.

**Table 1.** Potential function of exosomal microRNA in the pathogenesis of RA

Source	MicroRNA	Molecular effect	Ref.
Blood (patients)	miR-548a-3p↓	Increased proliferation and activation of pTHP-1 cells via the TLR4/NF-*κ*B signalling pathway	81
Plasma (patients)	miR-17↑	Decreased number of Treg cells because of suppressed TGFBR II expression	54
Serum (patients)	miR-6089↓	Increased proliferation and activation of macrophage-like THP-1 cells via TLR4 signalling	82
FLS (animal model)	miR-221-3p↑	Suppressed expression of Dkk2 in osteoblasts and differentiation of primary osteoblasts	64
FLS (patients)	miR-106↑	Decreased chondrocyte proliferation and migration, accelerated apoptosis and involvement of the RANKL/RANK/OPG axis via downregulation of PDK4 expression	67
Synovial fluid (patients)	miR-574-5p↑	Activated signalling through the TLR7/8 pathway, increased osteoclastogenesis and increased IFN-*α* and IL-23 mRNA levels in CD14+ monocytes	76

Dkk2, dickkopf WNT signalling pathway inhibitor 2; FLS, fibroblast-like synovial cells; IFN-*α*, interferon alpha; IL, interleukin; NF-*κ*B, nuclear factor kappa-Β; OPG, osteoprotegerin; PDK4, pyruvate dehydrogenase kinase 4; RANK, receptor activator of nuclear factor kappa-Β; RANKL, receptor activator of nuclear factor kappa-Β ligand; TGFBR II, transforming growth factor beta receptor 2; TLR, Toll-like receptor; Treg, regulatory T cells.

### EVs are involved in joint destruction

EVs, mainly synovial-derived EVs from RA patients, play a role in cartilage failure and joint impairment. An in vitro study has shown that RA-FLS-exos reduce osteoblast proliferation, mineralisation and differentiation (Ref. 63). Moreover, miR-221-3p levels were found to be upregulated in FLS-exos stimulated by mouse TNF, thereby negatively controlling the differentiation and mineralisation of skull osteoblasts in vitro (Ref. 64). In addition, exosomes from the synovial fluid of RA patients are detected more readily by the receptor activator of nuclear factor kappa B (NF-*κ*B)-ligand and present greater osteoclast formation potential than those of patients with osteoarthritis and ankylosing spondylitis (Refs 53, 65). Interestingly, circulating exosomes might exert the opposite effect. Compared with exosomes from healthy donors, circulating EVs from RA patients have been shown to inhibit osteoclast formation in vitro, indicating that circulating exosomes can exert a protective effect on bone resorption (Ref. 66). Moreover, RA-FLS-exos can suppress the proliferation and migration of chondrocytes and promote their apoptosis (Ref. 67). Several enzymes that promote matrix degradation, such as hexosaminidase D, are also related to EVs in RA. Early studies found that microparticles from T cells and monocytes could effectively induce pro-inflammatory mediators, chemokines and matrix-degrading enzymes in synovial fibroblasts, thereby aggravating angiogenesis, matrix degradation and cartilage damage in RA (Ref. 68). Collectively, EVs participate in the pathogenesis of RA by dysregulating bone and cartilage homoeostasis.

### EVs are involved in the Toll-like receptor signalling pathway

The Toll-like receptor (TLR) pathway is related to the pathogenesis of RA (Refs 69, 70). TLRs play an important role in triggering an immune response and inflammation (Ref. 71). TLR-mediated inflammation is believed to be involved in osteoclast-mediated bone erosion and joint vascularisation in RA (Refs 72–74). TLR activation influences the biological properties of released EVs. EVs can protect their ligands and even influence the response following ligand binding to TLRs. EVs protect and deliver their contents between cells in the extracellular environment (Refs 75–77), affecting intercellular communication.

The synovial tissue of RA patients has high levels of TLR3 and extracellular RNA (Ref. 78). EVs containing the TLR3 ligand polyinosinic-polycytidylic acid can efficiently transfer a limited amount of this content to synovial fibroblasts and reverse their natural pro-apoptotic behaviour. As a result, they may contribute to the formation of invasive synovial tissue capable of impairing articular cartilage (Refs 75, 79). High levels of miR-574-5p were detected in sEVs from both the synovial fluid and serum samples of RA patients. The overexpression of miR-574-5p in sEVs was reported to significantly increase osteoclastogenesis and elevate *IL-23* and *IFN-α* mRNA levels in CD14+ monocytes via TLR7/8 (Ref. 76). TLR7, which is elevated in RA, resides mainly in RA synovial fluid macrophages. miR-let-7b is a potential endogenous ligand of TLR7 (Ref. 80), and EVs containing miR-let-7b can reprogram M1 macrophages from RA primitive or anti-inflammatory macrophages through TLR7 ligation, thereby promoting the development of arthritis (Ref. 80). In addition, the expression of miR-6089 and miR-548a-3p, both of which can target TLR4 and thus inhibit the production of inflammatory cytokines IL-6, IL-29 and TNF-*α* in induced macrophage-like human acute monocytic leukemia cell lines (THP-1), was found to be significantly lower in exosomes from serum samples of RA patients (Refs 81, 82).

Oxidative stress is a hallmark of chronic diseases, including RA. Oxidative stress-derived EVs (stress EVs) were shown to be endogenous danger signals that can activate TLR4, leading to the expression of inflammation-related genes, such as *CCL24* and *IL-23*. Interestingly, inflammation resolution-related gene expression which cannot be induced by lipopolysaccharides was shown to be enhanced during the activation of stress EVs (Refs 77, 83). Furthermore, stress EVs could not induce tolerance in THP-1 macrophages to subsequent stress EV or lipopolysaccharide treatment, although macrophage stimulation by lipopolysaccharides could; this provides a new perspective on the involvement of stress EVs in the chronic aspect of these diseases (Refs 77, 84).

EVs, which are abundant in the plasma of RA patients, were demonstrated to stimulate NF-*κ*B signalling in transfected HEK 293T cells expressing TLR4 and its co-receptor MD-2, in addition to improving inflammation activity via their non-protein components (Ref. 83). Synthetic EVs constructed to mirror the composition of a phospholipid mixture of native EVs were shown to activate the TLR4 pathway under 15-lipoxygenase stimulation in vitro, suggesting that TLR4 senses oxidative stress mainly through stimulatory phospholipids rather than other components (Ref. 83). In addition, synergy between 15-lipoxygenase and secreted phospholipase A2 promotes inflammation through the formation of TLR4 agonists from EVs (Ref. 77). Phospholipase A2 activity is elevated in the synovial fluid of RA patients; secreted phospholipase A2 can promote K/BxN serum-induced arthritis, of which the severity is associated with TLR4 (Ref. 77). By serving as readily available substrates for lipid peroxidation and the preferred substrate for phospholipase A2, EVs can deliver lysophospholipids between cells and promote phospholipase A2 function (Refs 77, 85). Therefore, EVs might link oxidative stress with RA progression by facilitating ligand binding to TLR4 and subsequent downstream signalling.

These results indicate the potential role of EVs in protecting and shuttling their contents during the activation of TLR signalling. EVs can also influence the response of encapsulated ligands to their target receptors. In addition, TLR ligands such as polyinosinic–polycytidylic acid may regulate the composition of EV cargoes and coordinate their effects (Refs 86, 87). Thus, the interaction between EVs and TLR signalling specific to RA pathogenesis requires further study.

## MSC-EVs in RA

### Bone marrow mesenchymal stem cell-derived EVs

Bone marrow mesenchymal stem cells (BMSCs) can significantly improve symptoms of refractory patients and have shown sufficient immunomodulatory effects in two clinical studies (Refs 88, 89). These results are consistent with those of most arthritis models with BMSCs. Therefore, BMSC-derived EVs (BMSC-EVs), which share a similar effect to their parental cells, are a promising therapeutic agent for RA (Ref. 45).

Stella *et al*. first studied the role of BMSC-EVs in RA models and confirmed their sufficient efficacy in alleviating experimental RA by inhibiting T and B lymphocyte proliferation, as well as by inducing Treg and IL-10-expressing regulatory B cells in a dose-dependent manner. In this respect, the authors demonstrated that exosomes were more effective than microparticles (Ref. 45). The immunomodulatory effects of BMSC-EVs on macrophages have been shown in osteoarthritis models, both in vivo and in vitro (Refs 90, 91), although they have not been determined in RA models. In addition, BMSC-EVs play a role in bone and cartilage regeneration and angiogenesis (Refs 90, 92).

**Fig. 2.**
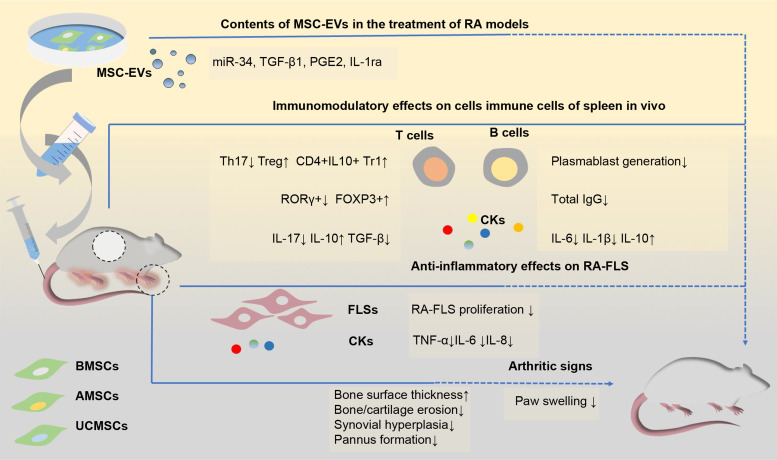
Schematic view of the potential mechanisms of mesenchymal stem cells-derived EVs in the treatments of rodent models of RA. EVs from different sources of MSCs show efficacy in the treatment of RA models. These EVs mainly show the immunosuppressive function of inhibiting T cell proliferation, downregulating Ig production and decreasing pro-inflammatory factors levels in vivo, thus attenuating clinical signs of paw swelling as well as histopathological indicators of bone and cartilage erosion and pannus formation. Several contents (including miR-34, TGF-*β*1 and IL-1ra) have been indicated to be associated with these functions. AMSC-EVs, adipose tissue mesenchymal stem cell-derived extracellular vesicles; BMSC-EVs, bone marrow mesenchymal stem cell-derived extracellular vesicles; CKs, cytokines; FLS, fibroblast-like synovial cells; FOXP3: forkhead box protein P3; Ig, immunoglobulin; IL, interleukin; IL-1ra, IL-1 receptor antagonist; miR-34, microRNA-34; MSC-EVs, mesenchymal stem cell-derived extracellular vesicles; PGE2, prostaglandin E2; RA, rheumatoid arthritis; ROR-*γ*, retinoic acid receptor-related orphan receptor *γ*; TGF-*β*: tumour growth factor beta; Th17, T helper 17; TNF-*α*, tumour necrosis factor alpha; Treg, regulatory T cells; Tr1, T regulatory type-1; UCMSC-EVs, umbilical cord mesenchymal stem cell-derived extracellular vesicles.

miRNAs contained in MSC-EVs are transferred to target cells and are the main effectors of MSC-EVs in various diseases (Ref. 93). FLSs, which act as effectors in RA, have been used as a therapeutic target in studies on BMSC-EV treatment for RA. Specifically, miR-34 in BMSC-EVs can reduce RA-FLS proliferation and inflammation by inhibiting the cyclin I/ATM/ATR/p53 signalling pathway (Ref. 35). In addition, several miRNAs downregulated in synovial tissue were found to be overexpressed in EVs and showed efficacy in treating inflammatory arthritis. One of them, miR-192-5p, can delay the inflammatory response in collagen-induced arthritis (CIA) rat models by targeting ras-related C3 botulinum toxin substrate 2 and regulating the immune response (Ref. 94). BMSC-exosome-derived miR-320 was found to specifically downregulate the chemokine ligand CXCL9 and inhibit the activation, migration and invasion of RA-FLSs (Ref. 95). Exosomal miR-150-5p regulates FLSs and inhibits angiogenesis by downregulating the levels of matrix metallopeptidase MMP14 and vascular endothelial growth factor (Ref. 96). A recent study demonstrated that the long non-coding RNA HAND2-AS1 could be combined with BMSC-EVs to suppress the tumour-like behaviour of RA-FLSs through the miR-143-3p/TNFAIP3/NF-*κ*B pathway, therefore impeding RA progression (Ref. 97) (Fig. 2).

Exosomes carrying specific RNAs can be assimilated by FLSs and effectively suppress inflammation during RA treatment, providing a novel potential cell-free therapeutic approach for RA. Although BMSC-EVs exert a positive effect on RA, their ability to improve immune regulation and anti-inflammatory mechanisms in RA have not been elucidated in much detail and warrant further investigation.

### Adipose tissue mesenchymal stem cell-derived EVs

Adipose tissue mesenchymal stem cells (AMSCs) have been shown to have the potential to treat RA. Human AMSCs (hAMSCs) regulate collagen-reactive T cell proliferation in RA patients, as well as their production of inflammatory and anti-inflammatory cytokines, such as IFN-*γ*, TNF-*α* and IL-10 (Ref. 98). A clinical trial evaluating the safety and tolerability of intravenous treatment in RA patients also suggested their potential clinical efficacy (Ref. 99). AMSC-derived EVs (AMSC-EVs) are thought to function similarly to AMSCs. Although several studies previously showed that AMSCs could ameliorate RA, studies exploring the role of AMSC-EVs in the treatment of RA have only emerged in recent years. Recent histological evidence has shown that EVs derived from wild-type mice ameliorated RA in a mouse model more effectively than EVs from IL-1Ra^−/−^ mice. The latter had no detectable expression of IL-1Ra, suggesting that exosomal IL-1Ra may be an effective indicator for the treatment of RA (Ref. 100). This will encourage the exploration of AMSC-EVs as an alternative treatment for RA.

Generally, AMSC-EVs exhibit an immunosuppressive effect on T cells (Refs 44, 101, 102). However, the effect varies among studies with different experimental settings and EV species sources. For example, in autoimmune encephalomyelitis (EAE) mice, Farinazzo *et al*. showed AMSC-EVs can partially inhibit T cells activation in vitro. However, they further demonstrated that this effect was insufficient in vivo (Ref. 42). Another study showed that treatment with allogenic AMSC-EVs can augment the percentage of Treg cells in the splenocytes of EAE mice (Ref. 103). In contrast, hAMSC-EVs were shown to exert no significant effect on Treg cells in the spleens of EAE mice (Ref. 104). In addition, hAMSC-EVs were shown to drive M2 macrophage polarisation, thus reducing the ability of macrophages to evoke inflammatory responses (Ref. 105). Interestingly, Rossana *et al*. demonstrated that only sEVs isolated from hAMSCs pre-activated with IFN-*γ* and TNF-*α* induced evident M2 macrophage polarisation (Ref. 106). Therefore, they suggested that the immunomodulatory effects of sEVs from AMSCs on macrophages may not be constitutive but rather induced by the inflammatory microenvironment (Ref. 106). Notably, María *et al*. indicated that culture medium derived from AMSCs can be more relevant than EVs in promoting an anti-inflammatory response (Ref. 88).

AMSC-EVs can also exert an immunomodulatory effect by delivering miRNAs. AMSC-EVs loaded with miR-10a (a relevant regulator of the CD4+ T cell subpopulation balance) can inhibit Th1 and Th17 responses, which indicates their potential therapeutic role as a delivery tool capable of precisely controlling immune cell differentiation (Ref. 107).

Considering that it is easier to choose autologous cells for cell-free therapy in large-scale populations, adipose tissue is a rich and safe source compared with other tissues. Proteomic analysis has indicated that AMSC-EVs are associated more tightly with immunomodulation-related proteins than BMSC-EVs (Ref. 108). In addition, AMSC-EVs have been shown to be more effective than BMSC-EVs at promoting cartilage and bone regeneration in a mouse model and represent a superior resource for cell-free therapy (Ref. 109). One study on osteoarthritis showed the capability of AMSC-EVs to reduce IL-*β*-mediated inflammation and cartilage degeneration (Ref. 110), further supporting their application in RA. Although, considering the contradictory nature of these results, the immunomodulatory function of EVs needs to be further investigated using RA models when thinking of their application to the treatment of RA.

### Umbilical cord mesenchymal stem cell-derived EVs

Based on the results of preclinical and clinical studies, umbilical cord mesenchymal stem cells (UCMSCs) have been suggested as a potential treatment for RA (Refs 111, 112). UCMSCs have been shown to be more effective at treating RA when cultured in a three-dimensional environment (Ref. 113), wherein they produced more exosomes, stimulated chondrocyte proliferation and migration and matrix synthesis, and inhibited cell apoptosis. Accordingly, the beneficial effect of UCMSCs in RA might be partly because of the exosome-mediated paracrine function of MSCs (Refs 113, 114).

A recent study has shown that human UCMSC-derived EVs (UCMSC-EVs) can ameliorate CIA by modulating T lymphocytes (Ref. 34), displaying greater efficacy than MSCs and methotrexate (Ref. 34). Previously, UCMSC-EVs were shown to restore the Th17/Treg balance, thus regulating inflammatory and anti-inflammatory factor secretion in blood samples of RA patients (Ref. 46), supporting their potential as a therapeutic candidate for RA. Interestingly, the effect of UCMSC-EVs at the transcriptional level varies depending on the setting, with FOXP3 protein and mRNA levels increased in the spleen and decreased in the joints of CIA rats. Considering that previous studies indicated that Tregs with impaired function were enriched in inflamed joints of RA patients (Refs 10, 11, 13), this inconsistency is assumed to represent a hypothetical mechanism for improving CIA. In a rat osteochondral defect model, exosomes from human Wharton's jelly derived mesenchymal stem cells could significantly promote the proliferation of chondrocytes and the polarisation of macrophages to the M2 phenotype, in addition to regulating inflammation of the joint cavity. Furthermore, sequencing and bioinformatics analysis suggested a possible functional effect of exosomal miRNAs in improving cartilage regeneration (Ref. 115). This evidence further suggests that UCMSC-EVs have a potentially beneficial effect on RA.

### Other stem cell-derived EVs

Exosomes from other types of stem cells have also been shown to affect immune activity and cartilage regeneration. Olfactory ecto-mesenchymal stem cells are a newly identified type of resident stem cell in the olfactory lamina propria. They have been found to inhibit the occurrence of arthritis and alleviate disease severity in an RA model. In vivo studies have further proven that they can regulate T cell responses and exert an immunosuppressive effect (Ref. 116). Recent studies on other autoimmune diseases have shown that the immunomodulatory effects of EVs derived from these cells encompass the regulation of Th1/Th17 and Treg cell responses (Ref. 43). Exosomes derived from gingival mesenchymal stem cells have been shown to exert immunosuppressive effects by regulating macrophage polarisation (Ref. 117). Specifically, these exosomes could promote the transformation from M1 to M2 macrophages and reduce the levels of pro-inflammatory factors TNF-*α*, IL-1*β* and IL-6, while significantly increasing levels of IL-10 by M1 macrophages in a high-lipid microenvironment (Ref. 118). In addition, exosomes from other types of MSCs are also involved in regeneration of the bone and cartilage. Recent studies on amniotic membrane mesenchymal cells and synovial mesenchymal stem cells have shown potential therapeutic effects in the treatment of osteoarthritis (Refs 119, 120). Furthermore, they can promote the maintenance and regeneration of bone tissue, enhance cell proliferation and suppress apoptosis, thereby preventing glucocorticoid-induced bone damage (Refs 121, 122). Considering the mechanisms that have been implicated in recent studies on the treatment of MSC-EVs in experimental arthritis, the functions reported above on immune activity and joint environment may also be involved in licensing these EVs to suppress autoimmune responses and inflammatory reactions in RA models.

Several studies have investigated the efficacy of MSC-EVs for the treatment of RA (Fig. 2, Table 2), providing a theoretical basis for further research. Notably, studies have shown that MSCs from different sources lead to distinct results in a disease environment, suggesting that there are differences in the therapeutic efficacy of EVs (Refs 119, 120). However, as only a few studies have directly compared EVs from different sources, more efforts should be directed towards comparing the various types of MSCs and the role of MSC-EVs in RA, eventually providing a foundation for future applications.

**Table 2.** Application of EVs in the treatment of RA

Source	Sub-type	EV properties (size, surface markers)	Disease model	Delivery mode	Biological effect	Ref.
BMSCs	EVs MPs Exosomes	MPs: >150 nm Sca-1, CD44, CD29 Exosomes: ~120 nm CD9 and CD81 Hsp70, Tsg101 and ALIX	CIA mouse DTH mouse	Intravenous injection for a week (on days 18 and 24) (250 ng)	Immunosuppressive effect of T and B lymphocytes Incidence and clinical scores↓ Bone degradation and erosion↓	45
BMSCs	sEVs	93 nm CD63+, CD81+ Calnexin	RA rat model	Single tail vein injection (75 μg/ml EVs)	Cyclin I negatively targeted by increasing exosomal miR-34a level Activation of the ATM/ATR/p53 signalling pathway TNF-*α*, IL-6 and IL-8 in synovial fluid↓ Synovial hyperplasia and inflammatory infiltration↓	35
AMSCs	MVs	200 nm	IL-1Ra^−/−^ mouse BALB/c mouse	Tail vein injection weekly for 4 weeks (5 μg EVs)	IL-1Ra contained in EVs transferred IL-1*β*，IFN-*γ*, TNF-*α* in serum↓ Synovial hyperplasia, pannus formation↓ Thickness of joints of paws ↓	100
hUCMSCs	sEVs	160 nm HSP70 and CD63 *β*-actin	CIA rats	Single tail vein injection (90 μg sEVs)	Immunomodulatory effect of T lymphocytes Synovial hyperplasia ↓ Synovial inflammatory cell infiltration ↓ Paw oedema and arthritis index↓	34
Human neutrophils	MVs	90% – 412 nm Mode frequency at 143 nm CD66b, annexin A1, TSG101, MRP8, MRP14, annexin V	K/BxN arthritis mouse model	Intra-articular injection unilateral knee for 3 days (3 × 10^4^, 5 μl final volume)	Sulphated glycosaminoglycan content loss prevented Cartilage degradation↓	133
G-MDSCs	sEVs	99.6 nm CD63 + calnexin	CIA mouse	I.p. injection (100 μg/injection) on days 18 and 24	Immunosuppressive effects of T and B lymphocytes Exosomal prostaglandin E2 stimulation of IL-10+ B cells Swelling of paws↓ Inflammatory cell infiltration and articular cartilage injury↓ Total Ig and anti-CII antibodies in sera↓	143

AMSC, adipose tissue mesenchymal stem cells; ATM, ataxia telangiectasia mutated; ATR, ataxia telangiectasia and radiation resistance gene 3-related; BMSC, bone marrow mesenchymal stem cells; CIA, collagen-induced arthritis; DTH, delayed-type hypersensitivity; G-MDSC, granulocytic myeloid-derived suppressor cells; HSP70, heat shock protein 70; hUCMSC, human umbilical cord mesenchymal stem cells; Ig, immunoglobulin; IL, interleukin; I.p., intraperitoneal; MP, microparticles; MRP, S100 calcium-binding protein; MVs, microvesicles; sEVs, small extracellular vesicles; TNF-*α*, tumour necrosis factor alpha; TSG101, tumour susceptibility gene 101.

## Non-MSC-derived EVs in RA

Early studies, including those on neutrophils and DCs, have shown that other cell therapies based on EVs can retard RA progression. Although the application of these EVs has rarely been investigated in recent years, their immunomodulation properties identified by accumulated research, combined with their efficacy as implicated by early studies suggest the value of further exploration in this field. Notably, the properties of EVs from DCs required for the treatment of RA are often derived by pre-modifying their parent cells.

### Dendritic cell-derived EVs

DC-derived EVs (DC-EVs) have been explored in the study of autoimmune diseases because of their parental cell properties. EVs from different DC subtypes exert a heterogeneous immune effect that includes immune stimulation and suppression. Studies have shown that the role of EVs from immature DCs (imDC-EVs) is strongly related to Treg cells. In a rat liver transplantation model, donor imDC-EVs induced the proliferation of recipient Tregs (Ref. 123). Another in vitro study showed that imDC-EVs could enhance the percentage of Foxp3 + CD4+ T cells and the transcription of *Foxp3* mRNA, while inhibiting the transcription of *IL-17A* mRNA under Th17 polarisation conditions (Ref. 124). Moreover, the exosomes of immature or inhibitory DCs have been shown to ameliorate the progression of experimental autoimmune myasthenia gravis by reducing the proliferation of acetylcholine receptor-reactive lymphocytes and the levels of pro-inflammatory factors (Ref. 125). It is worth noting that all imDC-EVs can induce T cell activation, but different imDC-EV subtypes can reverse the T cell response. Tkach *et al*. showed that EVs isolated using ultracentrifugation at different speed settings target distinct T cell subtypes (Ref. 126).

Making DCs tolerogenic, either via genetic modification or cytokine treatment, can render their EVs more immunosuppressive. The resulting exosomes could exert their function by directly or indirectly modifying the behaviour of endogenous immune cells, such as endogenous antigen-presenting cells and T cells, consequently affecting the entire body (Refs 127–130). Exosomes from TGF-*β*1-modified DCs can induce Foxp3 + CD4+ Tregs and lower the proportion of Th17 cells in the inflammatory site of inflammatory bowel disease (Ref. 131). Suppressive exosomes from DCs modified with immunomodulatory molecules, such as IL-10, TNF superfamily member Fas ligand (FasL), IL-4 and indoleamine 2,3-dioxygenase (IDO1) have been shown to mitigate the severity of RA in mouse models and suppress inflammation in a murine delayed-type hypersensitivity model. Therefore, these EVs exert both immunosuppressive and anti-inflammatory effects. MHC II, FasL, IL-4 and IDO1, as well as other molecules such as B7-1/2, are thought to be partially associated with these effects (Refs 127–130). In addition, DC-derived exosomes engineered to respond to reactive oxygen species through a new surface engineering method showed higher efficacy in the treatment of CIA, with prolonged circulation and enhanced accumulation in inflamed joints. This study further proved that the potential mechanism involved CD40, allowing EVs derived from tolerogenic DCs to mediate immunosuppressive effects during RA treatment (Ref. 132). In summary, these results provide another promising nanotherapeutic strategy for RA.

### Polymorphonuclear neutrophil-derived EVs

Polymorphonuclear neutrophil-derived EVs (PMN-EVs) are abundant in the synovial fluid of RA patients. PMN-EVs in the synovial fluid can directly interact with chondrocytes and regulate their homoeostasis, both in vivo and in vitro (Ref. 133). PMN-EVs can promote extracellular matrix deposition, prevent chondrocyte apoptosis and attenuate the secretion of prostaglandin E2 and IL-8, which are thought to be associated with extensive cartilage degradation in RA. Congruently, an in vivo study in a mouse model of K/BxN arthritis demonstrated that PMN-EVs prevent cartilage degradation (Ref. 133) (Table 2). PMN-EVs of RA patients are rich in annexin A1, a protein with tissue repair and pro-resolving properties, compared with those in plasma, which might help to explain the effect of those microvesicles on chondrocytes (Refs 133, 134). In addition, PMN-EVs are thought to affect macrophage–FLS crosstalk and prevent the excessive activation of adjacent FLSs (Ref. 135). Intriguingly, direct co-culture of neutrophils with chondrocytes can lead to chondrocyte death, whereas exposing them to neutrophil microvesicles provides protection, indicating that PMN-EVs might exert different effects than those of their parental cells (Ref. 133).

PMN-EVs from RA patients can modulate the macrophage phenotype, induce the release of the anti-inflammatory cytokine TGF-*β* in vitro and reduce the pro-inflammatory differentiation of macrophages more significantly in arthritic mice than in healthy controls (Ref. 135). This effect is believed to be partly dependent on the expression of phosphatidylserine and annexin A1 in microvesicles (Ref. 136). In addition, PMN-EVs can participate in the synthesis of lipid mediators of macrophages and increase the synthesis of resolvins, lipoxins and maresins, which are related to anti-inflammatory properties in arthritis (Refs 137, 138).

### Myeloid-derived suppressor cell-derived EVs

Myeloid-derived suppressor cells (MDSCs) represent a heterogeneous population at various stages of maturation and are more abundant in malignant, infectious, inflammatory and chronic diseases. MDSCs differ substantially between mice and humans but can be divided roughly into monocytic MDSCs and granulocytic MDSCs according to their corresponding surface markers and morphology (Ref. 139). MDSCs exert immunosuppressive effects on various target cells, but particularly affect the adaptive responses mediated by T cells (Refs 140–142). These suggest significant therapeutic effects in autoimmune diseases.

Recently, exosomes derived from MDSCs have been shown to attenuate the progression of arthritis in CIA mice (Table 2). Granulocytic MDSC-derived exosomes are thought to be more efficient than monocytic MDSC-derived exosomes, as they can suppress Th1 and Th17 cell differentiation and activation, promote B cells to secrete the anti-inflammatory cytokine IL-10 and decrease the proportion of plasma cells and follicular T helper cells. The bioactive molecules contained in exosomes, such as altered miRNA and prostaglandin E2, might explain these effects (Ref. 143). Notably, the control of neutrophil-derived exosomes in this study did not result in any relevant therapeutic effect (Ref. 144).

## Conclusions and future perspectives

EVs play an important role in the pathogenesis and treatment of RA. They mediate intercellular communication through the molecules they carry, such as mRNA, proteins and lipids. They participate in the dysfunction of immune activities, as well as bone and cartilage homoeostasis, contributing to the pathological changes in RA. The abnormal expression of miR-17, miR-574-5p and other molecules in EVs might assist in the diagnosis and treatment of RA. Exploring the exact origin of these EVs and their specific phenotypes could help develop novel approaches to diagnose and treat RA, such as diagnostic markers or EV-specific therapeutic drugs.

MSCs have been shown to be effective for the treatment of RA. The EVs derived from them are promising candidates for their similar properties to their parental cells in terms of immunomodulation and tissue regeneration. MSC-EVs are presumed to reduce the risk of side effects such as teratoma formation and immune rejection compared with that of viable cells, and several studies have elucidated the safety of EVs in therapeutic applications (Refs 145, 146). In addition, EVs from other sources, such as DCs, neutrophils and MDSCs, have also shown potential as novel cell-free treatment strategies for RA.

EVs can be designed to deliver specific mRNAs to the treatment of RA. They can also be harnessed to encapsulate small-molecule drugs owing to their natural properties and membrane-bound structure (Ref. 5). In addition, modifying parental cells in different culture environments can enhance the secretion and properties of their EVs. Future advancements in bioengineering will attune and optimise their characteristics and performance for various applications.

Nevertheless, challenges and roadblocks remain in this field. The precise classification, characteristics and properties of EVs have not been fully elucidated. Both the technology and methodology applied to EVs require refinement. Therefore, studies to identify the functional subgroups of EVs could be confounded and have yield contradictory results. To counteract such issues, standardised EV separation and quantification techniques should be prioritised to clarify the role and therapeutic potential of EVs for RA.
